# Clinical Value of FeNO for Pulmonary Hypertension Diagnosis in Patients with Acute Exacerbation of Chronic Obstructive Pulmonary Disease

**DOI:** 10.1155/2022/9924047

**Published:** 2022-01-28

**Authors:** Wei Guo, Ning Wang, Zhaobo Cui, Wenjing Liu, Shufen Guo, Xiaoya Yang, Yajing Liu, Liye Shao, Jing Wang

**Affiliations:** ^1^Department of Respiratory and Critical Care Medicine, Harrison International Peace Hospital Affiliated to Hebei Medical University, Hengshui 053000, China; ^2^Department of Critical Care Medicine, Harrison International Peace Hospital Affiliated to Hebei Medical University, Hengshui 053000, China

## Abstract

**Objective:**

To investigate the clinical value of fractional exhaled nitric oxide (FeNO) in the diagnosis of pulmonary hypertension (PH) in patients with acute exacerbation of chronic obstructive pulmonary disease (AECOPD).

**Methods:**

In this study, the medical records of AECOPD patients were retrospectively reviewed. The patients were divided into AECOPD and AECOPD + PH groups based on the absence or presence of PH. Moreover, FeNO and other indexes were compared between the two groups. The value of FeNO in diagnosing AECOPD with PH was determined using the ROC curve.

**Results:**

A total of 83 patients were enrolled (56 in the AECOPD group and 27 in the AECOPD + PH group). The level of FeNO was significantly lower in the AECOPD + PH group than in the AECOPD group (*P* = 0.022). Moreover, FeNO level (25.22 ± 8.45 ppb) was higher in the mild PH subgroup than in the moderate (16.64 ± 5.67 ppb, *P* = 0.005) or severe (11.75 ± 2.36, *P* = 0.002) PH subgroups. FeNO level was positively correlated with C-reactive protein in AECOPD patients while negatively correlated with brain natriuretic peptide in the AECOPD + PH group. ROC analysis showed that the optimal cutoff value of FeNO in the diagnosis of AECOPD with PH was 24.5 ppb.

**Conclusion:**

FeNO level at admission can act as an indicator for PH diagnosis in AECOPD patients.

## 1. Introduction

Chronic obstructive pulmonary disease (COPD) is a common respiratory disease and the fourth leading cause of death globally. Moreover, it may be the third leading cause of death in 2020 [1]. Presently, there are about 100 million COPD patients in China. The prevalence of COPD in people over 40 years old is as high as 13.7% [2]. COPD is a chronic airway inflammatory disease characterized by chronic cough, expectoration, and dyspnea. COPD patients commonly have acute exacerbations 0.5–3.5 times a year [3]. Acute exacerbation of chronic obstructive pulmonary disease (AECOPD) increases the inflammatory mediators, leading to airway mucosal congestion and edema, airway secretions, aggravates airway obstruction, increases alveolar pressure and pulmonary vascular resistance, and eventually leads to pulmonary hypertension (PH). AECOPD progression results in declined cardiopulmonary function, shorter survival, and higher mortality [4, 5].

AECOPD patients with PH may have serious cardiopulmonary function damage and a higher mortality rate. Moreover, PH severity significantly affects the prognosis of AECOPD patients [6, 7]. Some studies have shown that the quality of life of AECOPD patients can be significantly improved, and the mortality rate can be reduced if PH progression can be effectively delayed or prevented [7, 8]. Therefore, early diagnosis and treatment of AECOPD patients with PH are necessary for delaying disease progression and prolonging patients' life span.

American Thoracic Society (ATS) has recommended fractional exhaled nitric oxide (FeNO), as a noninvasive, simple, and safe method, for effective monitoring of airway inflammation and guide for the treatment of glucocorticoid in AECOPD patients [9, 10]. Studies have also shown that FeNO level is significantly increased in AECOPD patients but decreased in PH patients [11–13]. However, it is unknown whether pulmonary artery pressure affects elevated FeNO levels in AECOPD patients with PH. In this study, the concentration of FeNO in AECOPD patients with PH was assessed to evaluate its clinical significance in the diagnosis and treatment of AECOPD combined with PH.

## 2. Methods

### 2.1. Study Design and Participants

In this study, the medical records of AECOPD patients admitted in our department between January 2017 and May 2018 were retrospectively reviewed. Inclusion criteria included the following: patients aged ≥40 years, patients diagnosed with AECOPD based on 2017 Global Initiative for Chronic Obstructive Lung Disease (GOLD) guidelines [14], patients who are nonsmokers or quit smoking for at least half a year, patients with no systemic or have not inhaled corticosteroids and other drugs in the past month, and patients can cooperate with FeNO and pulmonary function examinations. Exclusion criteria were as follows: patients with allergic rhinitis, bronchial asthma, specific allergic disease, skin allergy, elevated eosinophils (normal reference range 0.02–0.52 × 10^9^/L), other lung diseases indicated by imaging examination, infection in other sites, other major systemic diseases (coronary atherosclerotic heart disease and diabetes), and other diseases that could affect FeNO results.

This study was conducted following the Declaration of Helsinki and was approved by the Institutional Ethics Committee Board of Harrison International Peace Hospital (No. 2017-1-006).

### 2.2. Procedures and Assessments

The patients were divided into AECOPD and AECOPD combined with PH (AECOPD + PH) groups based on the absence or presence of PH. Patients in the AECOPD + PH group were further divided into mild, moderate, and severe subgroups based on the status of pulmonary artery pressure (mild PH, pulmonary arterial systolic pressure (PASP) ≥35 mmHg to <45 mmHg; moderate pulmonary hypertension, PASP ≥45 mmHg to <60 mmHg; severe PH, PASP ≥60 mmHg). 

FeNO levels were measured after emergency room admission and before symptomatic treatment using an electrochemical breath analyzer (Shangwo Medical Electronics, Wuxi, China) by the same testing team. The measurement was conducted based on the FeNO operation standard recommended by ATS and European Respiratory Society (ERS). The patients were not allowed to eat 2 hours before the FeNO measurement and not allowed to drink 12 hours before the measurement. FeNO detection was performed before the pulmonary ventilation function test to avoid affecting the final FeNO measurement result due to airway tension change caused by forced exhalation. The patients kept breathing steadily and smoothly for at least two breath cycles. The patients inhaled NO-free gas to their total lung capacity and exhaled at a constant airflow of 50 mL/s for 10 s. Patients were asked to tightly hold the filter with their mouths to ensure no air leakage at the corner of the mouth or blockage of the filter. The FeNO analyzer was used to automatically record the FeNO concentration at a stable condition for at least 3 s. FeNO levels were measured thrice for each patient with a difference of not more than 10%.

A pulmonary function analyzer was used to evaluate the pulmonary function-related parameters. All measurements were performed in triplicate. Forced expiratory volume in the first second (FEV1) was graded based on GOLD guidelines. Grades 1, 2, 3, and 4 indicated FEV1 ≥80%, 50% ≤FEV1 <80%, 30% ≤FEV1 <50%, and FEV1 <30%, respectively.

Echocardiography was used to measure the peak velocity of tricuspid regurgitation (V). The PASP was estimated based on the guidelines for diagnosis and treatment of PH published by ESC/ERS in 2015 [15].

### 2.3. Statistical Analysis

SPSS software (version 21.0) was used for all statistical analyses. Variable data with normal distribution are expressed as mean ± standard deviation (*x* ± *s*), while attribute data are expressed as percentages. An independent sample *t*-test was used to compare the mean values of the two groups. ANOVA was used to compare PASP and FeNO levels of patients with different PH grades. The LSD *t*-test was used for comparison between groups. The *χ*^2^ test was used to compare the data determined based on GOLD classification and mechanical ventilation rate between the two groups. Pearson correlation analysis was used for correlation analysis. A receiver operating characteristic (ROC) curve was used to analyze the diagnostic value of FeNO for PH diagnosis in AECOPD patients. *P* < 0.05 was considered a statistically significant difference.

## 3. Results

### 3.1. Patient Baseline Characteristics

In this study, 83 eligible patients were enrolled (56 in the AECOPD group and 27 in AECOPD + PH group). Baseline characteristics are given in Table 1. Age, gender, smoking rate, GOLD grade, and arterial partial pressure of carbon dioxide (PCO_2_) were not significantly different between the two groups (*P* > 0.05). However, arterial blood pH, arterial oxygen partial pressure (PO_2_), C-reactive protein (CRP), brain natriuretic peptide (BNP), length of hospital stays, and invasive mechanical ventilation rate were significantly different between the two groups (*P* < 0.05).

### 3.2. FeNO Levels

The mean levels of FeNO in AECOPD and AECOPD + PH groups were 33.14 ± 13.38 ppb and 18.78 ± 7.94 ppb, respectively. FeNO level was significantly lower in the AECOPD + PH group than in the AECOPD group (*P*=0.022).

### 3.3. FeNO and PASP Levels of Different Grades of PH in the AECOPD + PH Group

**Figure 1:**
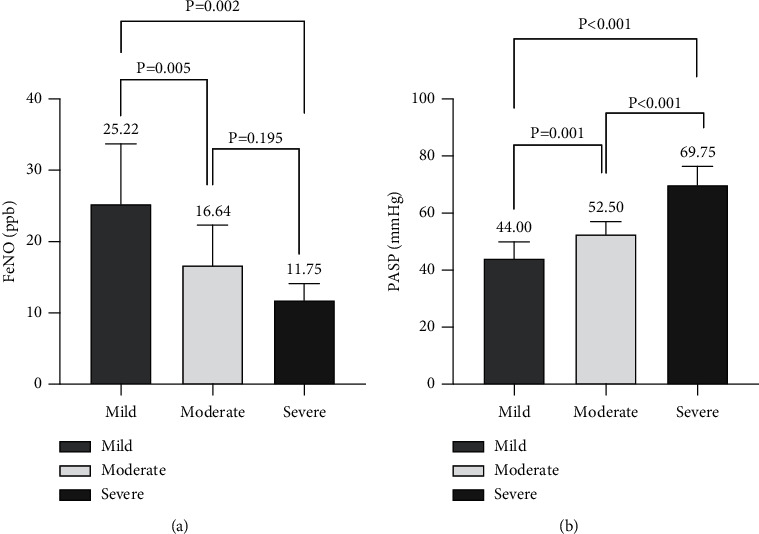
(a) FeNO and (b) PASP levels in patients with mild, moderate, or severe PH in the AECOPD + PH group. FENO, fractional exhaled nitric oxide; PH, pulmonary hypertension; PASP, pulmonary arterial systolic pressure.

The patients in the AECOPD + PH group were further divided into three subgroups based on PH severity (9 patients in the mild subgroup, 14 patients in the moderate subgroup, and 4 patients in the severe subgroup). FeNO levels (25.22 ± 8.45 ppb) were significantly higher in the mild subgroup than in the moderate (16.64 ± 5.67 ppb, *P*=0.005) and severe (11.75 ± 2.36, *P*=0.002) subgroups (Figure 1(a)). PASP levels were 44.00 ± 5.87 mmHg, 52.50 ± 4.50 mmHg, and 69.75 ± 6.65 mmHg in mild, moderate, and severe subgroups, respectively (Figure 1(b)). FeNO and PASP levels were significantly different among the subgroups (mild vs. moderate, *P*=0.001; mild vs. severe, *P* < 0.001; moderate vs. severe, *P* < 0.001).

### 3.4. Relationship between FeNO vs. BNP and CRP

**Figure 2:**
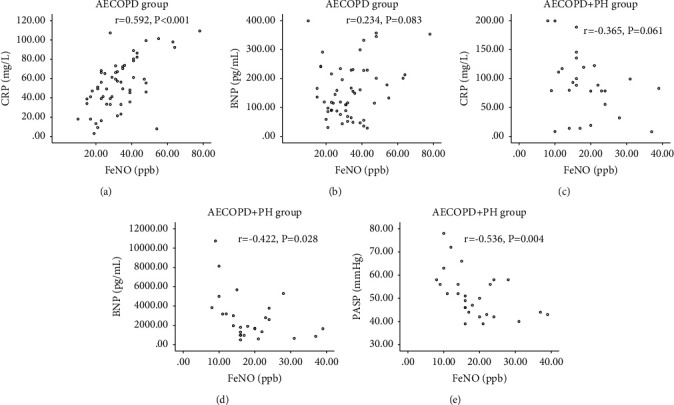
Relationship between FeNO vs. BNP, CRP, and PASP in AECOPD patients with or without HP. (a) Relationship between FeNO and CRP in AECOPD patients. (b) Relationship between FeNO and BNP in AECOPD patients. (c) Relationship between FeNO and CRP in AECOPD + PH patients. (d) Relationship between FeNO and BNP in AECOPD + PH patients. (e) Relationship between FeNO and PASP in AECOPD + PH patients. FENO, fractional exhaled nitric oxide; PH, pulmonary hypertension; BNP, brain natriuretic peptide; CRP, C-reactive protein; PASP, pulmonary arterial systolic pressure.

FeNO level was positively correlated with CRP in the AECOPD group (*r* = 0.592, *P* < 0.001; Figure 2(a)). However, FeNO level was not correlated with BNP (*r* = 0.234, *P* = 0.083; Figure 2(b)). Moreover, FeNO level was not significantly correlated with CRP in the AECOPD + PH group (*r* = −0.365, *P* = 0.061; Figure 2(c)). However, FeNO was negatively correlated with BNP (*r* = −0.422, *P* = 0.028; Figure 2(d)). Moreover, FeNO level was negatively correlated with PASP in AECOPD + PH patients (*r* = −0.536, *P* = 0.004; Figure 2(e)).

### 3.5. Diagnostic Value of FeNO for PH

**Figure 3:**
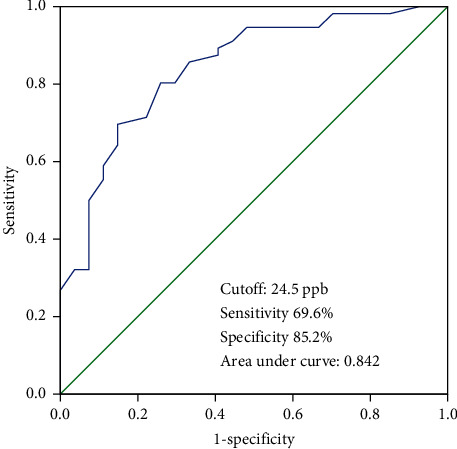
ROC curve showing the diagnostic value of FeNO level for PH in AECOPD patients.

The ROC curve was used to analyze the diagnostic value of FeNO level in AECOPD patients with PH. The area under the curve (AUC) was 0.842 (95% confidence interval (CI) = 0.752–0.932), and the optimal cutoff value of FeNO in the diagnosis of AECOPD with PH was 24.5 ppb (sensitivity, 69.6% and specificity, 85.2%; Figure 3). These results indicate that FeNO level can diagnose AECOPD patients with PH.

## 4. Discussion

In this study, FeNO level was positively correlated with CRP level in AECOPD patients, while it was negatively correlated with BNP in AECOPD patients with PH. The optimal cutoff value of FeNO in PH diagnosis in AECOPD patients was 24.5 ppb, indicating that FeNO can be used to diagnose AECOPD patients with PH.

Moreover, FeNO level was significantly lower in the AECOPD + PH group than in the AECOPD group, indicating that PH significantly affects FeNO level. FeNO levels were significantly lower in moderate and severe pH subgroups than in the mild PH subgroup. However, FeNO level was not significantly different between the moderate and severe groups. The stabilization of FeNO concentration may be due to the continuous degeneration and necrosis of endothelial cells with the prolongation of the course of AECOPD, thus decreasing the number of endothelial cells producing FeNO, which gradually reaches the peak. However, further studies with larger sample sizes are needed to confirm the results.

Gupta et al. found that AECOPD patients with relatively low FeNO levels had a longer hospital stay [16]. Herein, AECOPD patients with PH had a longer hospital stay than those without PH. Therefore, low FeNO levels in AECOPD patients may be due to PH.

Correlation analysis revealed that FeNO level was negatively correlated with PASP in AECOPD + PH patients. ROC curve analysis showed that the best cutoff value, sensitivity, and specificity of FeNO in diagnosing AECOPD patients with PH was 24.5 ppb, 69.6%, and 85.2%, respectively. Therefore, FeNO level of AECOPD patients less than 24.5 ppb could indicate the presence of PH. Moreover, FeNO may be a new potential indicator for identifying PH in AECOPD patients due to the invasive nature of the floating catheter and the subjectivity of echocardiography.

CRP is a polysaccharide produced by human serum during acute inflammation. AECOPD patients have increased CRP levels [17], while AECOPD patients with PH have significantly increased CRP levels [18]. Herein, FeNO level was positively correlated with CRP in AECOPD patients without PH. However, FeNO level was not correlated with CRP in AECOPD patients with PH. This result may be due to different effects of AECOPD and PH on FeNO level, indicating that PH affects FeNO level as an indicator of exhaled inflammation.

BNP is a vasoactive substance with many functions, such as vasodilation, natriuretic, and reducing vascular resistance. Previous studies have shown that BNP levels are significantly higher in AECOPD patients with PH than those without PH [19]. Besides, BNP levels are positively correlated with PASP in AECOPD patients [20]. Herein, correlation analysis revealed that FeNO is not correlated with BNP in AECOPD patients without PH. However, FeNO was negatively correlated with BNP in AECOPD patients with PH. However, further studies with large samples are needed to assess the correlation between FeNO level vs. CRP and BNP.

However, this study has some limitations. First, some patients with mild symptoms may go to the community for treatment; in contrast, some patients with severe symptoms may not undergo the corresponding examination, leading to bias in case selection. Second, the floating catheter of the right heart artery is the gold standard of PASP detection. In this study, echocardiography was used to estimate pulmonary artery pressure due to its invasive. However, echocardiography may introduce bias due to subjectivity. Third, the number of patients enrolled was relatively small. Fourth, there was no priori power analysis before the initiation of this study and no follow-up after this study. Fifth, the pathomechanism of AECOPD and PH, particularly processes reducing NO production, is different. Therefore, it is unknown whether FENO can predict AECOPD in patients with PH, and thus, further studies are needed. Further studies with large samples are needed to assess whether FeNO level can guide the treatment and predict the prognosis of AECOPD patients with PH.

In conclusion, FeNO can be used to diagnose AECOPD patients with PH. Therefore, this study further promotes understanding of airway inflammatory diseases and the diagnosis and treatment of AECOPD patients.

## Figures and Tables

**Table 1:** Baseline characteristics.

	AECOPD (*n* = 56)	AECOPD + PH (*n* = 27)	*t*/*χ*^2^	*P* value
Male	34 (60.7%)	15 (55.5%)	0.200	0.654
Age (years)	67.91 ± 6.95	68.11 ± 6.74	−0.124	0.901
Smoker	35 (62.5%)	19 (70.3%)	0.496	0.481
GOLD grade			2.743	0.254
Grade 1	0	0	—	—
Grade 2	29 (51.8%)	10 (37.0%)	—	—
Grade 3	26 (46.4%)	15 (55.6%)	—	—
Grade 4	1 (1.7%)	2 (7.4%)	—	—
PASP (mmHg)				
Mild	—	44.00 ± 5.87	—	—
Moderate	—	52.50 ± 4.50	—	—
Severe	—	69.75 ± 6.65	—	—
Arterial blood pH	7.35 ± 0.05	7.31 ± 0.09	2.339	0.025
PO_2_ (mmHg)	60.10 ± 6.99	53.76 ± 6.52	3.957	<0.001
PCO_2_ (mmHg)	56.76 ± 15.64	64.40 ± 21.04	−1.859	0.067
CRP (mg/L)	54.26 ± 26.31	90.24 ± 54.47	−3.254	0.003
BNP (pg/ml)	156.32 ± 93.25	8721.00 ± 8128.70	−3.707	0.001
Hospital stays (days)	8.64 ± 2.26	13.22 ± 3.29	−6.535	<0.001
Invasive mechanical ventilation	5 (8.9%)	7 (25.9%)	4.256	0.039
FeNO (ppb)	33.14 ± 13.38	18.78 ± 7.94	5.430	0.022

Data are shown in (%) or mean ± SD. PO_2_, arterial oxygen partial pressure; PCO_2_, arterial partial pressure of carbon dioxide; CRP, C-reactive protein; BNP, brain natriuretic peptide.

## Data Availability

The data used to support the findings of this study are available from the corresponding author upon request.

## Abbreviations

PO_2_: Arterial oxygen partial pressure
PCO_2_: Arterial partial pressure of carbon dioxide
CRP: C-reactive protein
BNP: Brain natriuretic peptide
AECOPD: Acute exacerbation of chronic obstructive pulmonary disease
FeNO: Fractional exhaled nitric oxide
GOLD: Global Initiative for Chronic Obstructive Lung Disease
PH: Pulmonary hypertension
FEV1: Forced expiratory volume in the first second
ROC: Receiver operating characteristic.
